# The Roots of Bioinformatics in Protein Evolution

**DOI:** 10.1371/journal.pcbi.1000875

**Published:** 2010-07-29

**Authors:** Russell F. Doolittle

**Affiliations:** 1 Department of Chemistry & Biochemistry, University of California, San Diego, La Jolla, California, United States of America; 2 Department of Molecular Biology, University of California, San Diego, La Jolla, California, United States of America; Philadelphia, United States of America

## Introduction

Bioinformatics as a formal discipline came of age in the late 1980s, greatly stimulated by the 1989 Human Genome Initiative. The roots of the field go back several decades earlier, however, to an era when computers were not needed to manage the data. In this personal reflection, I review the confluence of events beginning in the 1950s that brought a number of fields together in a common pursuit. Particularly, I offer some comments about early amino acid sequence comparisons, the results of which revealed so much about evolution, and how the computer became necessary only when the number of known sequences began to grow exponentially.

Many other authors have already recorded their thoughts on the evolutionary roots of bioinformatics in accounts that are doubtless more thorough and balanced than can be recorded in this brief personal reflection ([1], [2], inter alia). All are in agreement about certain pivotal events that were true milestones: the double-helix model of DNA, the first determination of the amino acid sequence of a protein, and the conceptual linking of DNA sequences and protein sequences. My plan is to expand on some related matters with the hope of providing some additional background on those early scenes.

## Sequences

Sequences, the simple order of individual units in biological polymers, are at the heart of bioinformatics, and the search for relationships among them and the reconstruction of their histories has arguably proved the most informative of biological inquiries. Today dozens of giant data banks store what seem to be countless numbers of nucleic acid and protein sequences. But there was a time, only 50 or 60 years ago, when hardly any sequences were known at all. Nonetheless, there were those who already appreciated that the web of all life would eventually be reconstructed on the basis of sequence data alone. There was an obligatory progression of events, beginning with chemistry, then biology, and, finally, the need for computers.

Among the technological advances that made sequence determinations possible, two are extremely notable: the introduction in the 1940s of paper chromatography as a simple tool for identifying amino acids and their derivatives [3], for one, and the use of suitable chemical reagents that reacted (more or less) exclusively with amino groups, for another—particularly an amino-tagging reagent by Sanger [4] and an amino acid-labilizing reagent by Edman [5]. Some important details of their seminal and unique contributions need to be described here, however briefly.

### Chemistry

It must be difficult for a young scientist today to imagine how primitive circumstances were in the mid-20th century. The effort needed to determine even a short amino acid sequence was more than considerable; it was daunting (some of that tedium may carry through in the following description).

Typically, the first step in determining the sequence of a peptide or protein was to establish its amino acid composition. It was well known that heating a protein or peptide with strong aqueous acid broke the bonds between the constituent amino acids (unhappily, glutamines and asparagines were changed into glutamic and aspartic acids in the process, and a few other amino acids like tryptophan damaged). The resulting hydrolysate could be spotted on a large piece of filter paper and separation of the various amino acids obtained by letting an organic solvent creep over the paper, partitioning the amino acids according to their relative solubilities in one phase or the other. The locations of the amino acids could be found by staining the dried paper with ninhydrin, a compound that gave a blue color with amino groups.

After a preliminary amino acid composition was in hand, the next step was to break the protein or peptide into smaller pieces (the “divide and conquer” strategy). The simplest method was to use partial acid hydrolysis, taking advantage of the fact that bonds next to some amino acids break more easily than others. The other popular option was to use proteolytic enzymes like trypsin or chymotrypsin. In either case, the peptide fragments were purified, often by paper chromatography, and their individual amino acid compositions determined.

Indeed, one reason that protein sequences were attacked first, rather than RNA or DNA, was because there were 20 different amino acids, and a random, partial hydrolysis of a polypeptide chain could give rise to smaller peptides with unique compositions. The logistics of the same approach for a polymer made of only four different things was impossible to contemplate.

### More Chemistry

The Sanger reagent, fluorodinitrobenzene (FDNB), had several important features. First, the bond between it and the tagged amino acid was resistant to acid hydrolysis; second, the derivatized amino acid was sufficiently non-polar that it could be extracted from the acid hydrolysate with an organic solvent like ether; and finally, the derivatives were bright yellow and could be readily identified by paper chromatography. The operation could be conducted on the starting peptide or protein, as well as on the fragments generated by various means. It was a slow and arduous process and very much limited to small-ish proteins.

The Edman reagent, phenylisothiocyanate (PheNCS), utilized a completely different strategy. A related compound phenylisocyanate (PheNCO) had previously been shown to be a labilizing agent that could tag and release an amino acid from the amino-terminus of a peptide and had been used successfully on a tripeptide as long ago as 1930 [6]. Edman's PheNCS was much superior, however, as the sulfur atom was much more favorably disposed to the second step of the operation, a rearrangement that led to the separation of the terminal amino acid from the parent peptide or protein in anhydrous acid, conditions that left the remaining peptide bonds intact. As a result, the operation could be repeated over and over again, alternating between a coupling reaction at high pH and cleavage in dry acid, liberating one amino acid at a time from the amino-terminal end. The cleaved residues, labeled as they were with the PheNCS, could be extracted with an organic solvent and, once again, identified by paper chromatography.

Because the coupling and cleavage at each step was never 100 percent complete, the operation tended to get out of phase and was no longer informative after several cycles. Additionally, the parent peptide tended to wash away during the repeated extractions, imposing a further limit on how many cycles could be conducted successfully. Nonetheless, it was an elegant method, even allowing for the typical procedure being limited to one amino acid cycle per day. Moreover, procedures that combined the Sanger and Edman approaches were devised, and these speeded up determinations significantly.

Column partition methods for separating peptides and amino acids were also being developed during this period, and the introduction of an automatic amino acid analyzer in the late 1950s was much heralded by the claim that a single analysis could now be performed in as little as 24 hours [7]!

### Biology

From the sequence perspective, the 1950s was largely a decade of polypeptide hormones, several of which exhibited distinct similarities to each other. The amino acid sequence of bovine (cattle) insulin, which is composed of two chains totaling 51 residues, was completed in 1955 [8], as was pig corticotropin, a single polypeptide chain of 39 residues [9]. As was the case for insulin, corticotropins from several species revealed a variability restricted to one small region.

Once the technology was developed for determining protein sequences, choices had to be made about which proteins to study. The necessary restrictions were that they be abundant, small, and easy to purify (and fundable). As it happened, a relatively small group of such proteins was able to provide insights into the two subjects of most interest to evolutionists, which were intra- and inter-species sequence variability, for one, and gene duplications and the evolution of new proteins, for the other.

The most popular proteins for study in the 1950s were—in order of increasing size—cytochome c, ribonuclease, hemoglobin, and the serine proteases. The first of these to be completed, and the first of more than a hundred residues, was cytochrome c [10].

It may seem a meager list today, but this small cast set the stage for all that was to follow. Hemoglobin was probably the most illuminating, providing the most useful data on several fronts. By this time it was known that most vertebrate hemoglobins were composed of two pairs of subunits the size of myoglobin, and these were genetically endowed in the fashion of one gene, one polypeptide chain.

The discovery in 1949 that an apparent single amino acid replacement in hemoglobin could lead to a disease in which red blood cells became sickle shaped was a blockbuster [11]. The impact was almost as great 9 years later when Vernon Ingram showed that the particular replacement was a valine for a glutamic acid [12]. In line with the techniques of the day, Ingram had first digested normal and sickle cell hemoglobins with trypsin and then used a combination of paper chromatography and electrohoresis to make a two-dimensional map of the resulting peptides. A comparison of maps made from normal and sickle cell hemoglobins showed that only one of the spots had shifted its position, and the amino acid composition of that peptide showed that the change was from a glutamic acid in the normal hemoglobin to a valine in hemoglobin S.

The combination method of paper electrophoresis and chromatography, which Ingram called “fingerprinting,” was quickly taken up by other labs for identifying changes in other variant hemoglobins, a large number of which had been identified clinically. Fingerprinting was also a simple way for comparing hemoglobins and other proteins from different species, and several other laboratories promptly undertook such studies. Emile Zuckerkandl and Dick Jones, working in Linus Pauling's laboratory, began a study of hemoglobins from different species by this method [13], and workers in Chris Anfinsen's group began charting differences in various animal ribonucleases [14]. By this time, also, full determinations of cytochrome c sequences from several sources were under way in several laboratories.

In 1959, Anfinsen's book *The Molecular Basis of Evolution* appeared [15]. This slender volume provided some basic paleontology and genetics as background, as well as the rudiments of DNA and protein structure, including a few simple sequence comparisons of hormones and partially sequenced proteins. Anfinsen coupled his discussions with some bold pronouncements about how DNA sequences must be correlated with amino acid sequences. Even though the genetic code was yet to be deciphered, he conjured up a fictitious set of base triplets and showed how single base substitutions in the gene for the human hemoglobin β chain could change the wild type glutamic acid into the valine found in hemoglobin S and how another single base change at the same position could yield the lysine found in hemoglobin C.

### More Biology

In 1957, Harvey Itano wrote a lengthy review about the genetics of hemoglobins [16], cataloging a wide variety of single amino acid replacements that occurred in “variants,” including hemoglobins S, C, and so forth. He went on to propose that there must be distinct genes for several globin polypeptides, including fetal and embryonic types, and that these must be the result of a series of gene duplications. By then, it was possible to separate the different polypeptide chains of hemoglobin by ion exchange chromatography, and the several adult, fetal, and embryonic forms became available in pure form. Even before their sequences were complete, it was clear they were the result of the phenomenon casually called “gene duplication.”

Although the duplication of genetic material was first observed during the 1920s by geneticists studying fruit flies [17], it wasn't until the 1950s that the concept of gene duplication as a force in evolution was fully recognized [18], [19]. By 1961, the laboratories of Ingram [20] at MIT and Braunitzer [21] in Germany had finished the sequences of both the α and β chains of hemoglobin; they were found to be 45% identical, leaving no doubt of their common ancestry.

Other examples of gene duplication were being found. Indeed, in the mid-1950s, several different polypeptide hormones were already known to have similar amino acid sequences, beginning with du Vigneaud's classic work on the nonapeptides vasopressin and oxytocin [22]. Any thought that this was a phenomenon limited to small peptides was dispelled in 1964 when Walsh and Neurath determined the sequences of trypsinogen and chymotrypsinogen from bovine pancreas, each of which was well over 200 residues in length. After suitable alignment, they were found to be 38% identical [23].

In some cases, proteins were being found that had obviously been elongated by tandem duplication within genes, including a bacterial ferredoxin [24], [25] and the various chains of immunoglobulins [26], [27]. On another front, distinct changes of function were being observed for related proteins. For example, the milk protein lactalbumin was found to be related to the enzyme lysozyme [28], but its new role was not that of an enzyme.

It is significant that none of these relationships needed a computer to be discovered.

## A Graduate Student Perspective

All of these endeavors, chemical and biological, were well under way when I began graduate school in 1957. My awareness of them began almost immediately in an introductory biochemistry course taught by George Wald. Wald was a Harvard professor renowned for his stimulating lecturing style. He would personalize every observation. “Sanger introduced the use of fluorodinitrobenzene, a reagent that reacts with amino groups and which imparts a yellow color to the terminal amino acid of a protein,” and “du Vigneaud determined the structure of oxytocin and vasopressin, and they differ in only two of their nine amino acids.”

He always managed to work the very latest findings into his lectures, every new discovery having an individual's name associated with it: “Sanger has completed the sequence of insulin from five species, and they differ at only three places among their 51 amino acids.” “Hans Neurath has shown how trypsinogen is converted into trypsin,” and “Emil Smith is working on the sequence of papain,” and “Moore and Stein have almost worked out the sequence of ribonuclease, which has 124 amino acids.”

It is remarkable how much Wald was able to pack into these 50-minute lectures using only a chalk board. It was an era when students religiously took notes in spiral ring notebooks, one benefit of which is that I have mine beside me as I write this reflection more than a half century later.

As it happened, I ended up in a laboratory studying blood proteins and blood clotting, and it was natural to be thinking about these proteins in light of all the new discoveries. Why did the proteins of different organisms have different sequences? And, especially, where did new and different proteins come from?

It was a grand time to be a student. The curtain was rising on the Greatest Show on Earth. The entire drama of life was being revealed by connections between DNA and proteins. It was proposed that there was a genetic code, already presumed to be triplet in nature, by which the sequences in DNA were translated into sequences of amino acids.

Any doubts about this being the case were erased in the summer of 1961, at which time the International Congress of Biochemistry was being held in Moscow. Only a relatively few intrepid American scientists were able to make the journey “behind the Iron Curtain,” but immediately upon their return the electrifying news of the finding by Nirenberg and Matthai that had been reported at the meeting [29] spread quickly through the biochemical community by telephone. Almost half a century later, I can still remember a friend calling me with the terse message: “Poly-U makes poly-Phe.” There was no doubt now that the genetic code would be broken.

### Fibrinopeptides

During my graduate years I learned how to purify proteins and peptides and began work on what was known in those days as the “species specificity problem,” in which a pair of interacting proteins from a given species seemed mutually adapted and were more effective than when either was reacted with the corresponding partner from another species. The protein pair I was studying was thrombin and fibrinogen [30]. Thrombin acts by cleaving a pair of peptides, called fibrinopeptides, from fibrinogen, after which a fibrin clot forms spontaneously.

During the course of this work I had purified fibrinopeptides from several species and determined their amino acid compositions by paper chromatography. Even at this stage, it was apparent that the fibrinopeptides differed greatly from species to species, a reflection of their very simple functional needs. Certainly they seemed to be changing much more rapidly than rates inferred from preliminary data from other proteins like hemoglobin or cytochrome c.

As it happened, I learned that workers in Sweden were already sequencing fibrinopeptides by the Edman method. Wisely, I wrote and asked if I could join them, and with the aid of a National Institutes of Health (NIH) postdoctoral fellowship, set off to Stockholm. By good fortune, Birger Blomback had just returned from visiting Pehr Edman's laboratory in Australia, and I was able to learn the latest wrinkles in how to execute stepwise degradations. For the most part, the fibrinopeptides were marvelously amenable to that method, the only exceptions being when the amino-termini were blocked (an unfortunately common occurrence in fibrinopeptides B). One feature that allowed successful consecutive degradation of (unblocked) fibrinopeptides was that they all had carboxy-terminal arginine, the very polar guanidine group holding the parent peptide in the aqueous phase while the derivatized terminal residues were being extracted with organic solvents. As a result, even at the standard pace of one residue per day per peptide, we were able to completely sequence peptides from several species rather quickly [31].

And it was exciting! One couldn't wait to see what the next residue was going to be. There were simple but profound questions to be answered: why should a sheep have an alanine at a position in some protein where a cow had a threonine? Did it matter? Were these really “neutral” changes? How did such changes become “fixed”?

In order to answer these questions I was forced to bone up on a good deal of classical biology, especially with regard to the phylogenetic relationships of the animals we were studying. Quite by chance, five of the first seven species we worked on belonged to the same mammalian order. Pigs, sheep, goats, reindeer, and domestic cattle are all artiodactyls. Even though they were closely related, there were numerous changes among them (sheep and goat were identical, however).

In the spring of 1963 I wrote a long letter to George Gaylord Simpson, the eminent paleontologist, asking his opinion about how long ago in millions of years these various creatures had common ancestry. He answered immediately, and his hand-scrawled estimates served as abscissa points in a plot of amino acid replacements versus time in our article in *Nature* [31].

Our correspondence continued. Simpson, who was very interested in our data, very reasonably tried to curb some of my enthusiasm for reclassifying creatures on the basis of a small number of amino acid replacements. Simpson also set me straight on some estimates of generation times for these animals. He was also rather negative about putting any stock in the kinds of change involving “spacers” that I was touting. I had defined “spacers” as residues occurring at fast changing positions that seemed to tolerate a variety of different amino acids and whose function seemed merely to occupy space between other more important residues.

On the other hand, Simpson was certainly enthusiastic about the sequence approach in general, even though he ended one of his letters with the following cautionary lines about sequence comparisons:

“It seems to me that the subject is now still in a pioneering and pilot-stage, and that firm conclusions cannot yet be expected–It might eventually do more harm than good to expect them at this stage.”

**Figure 1:**
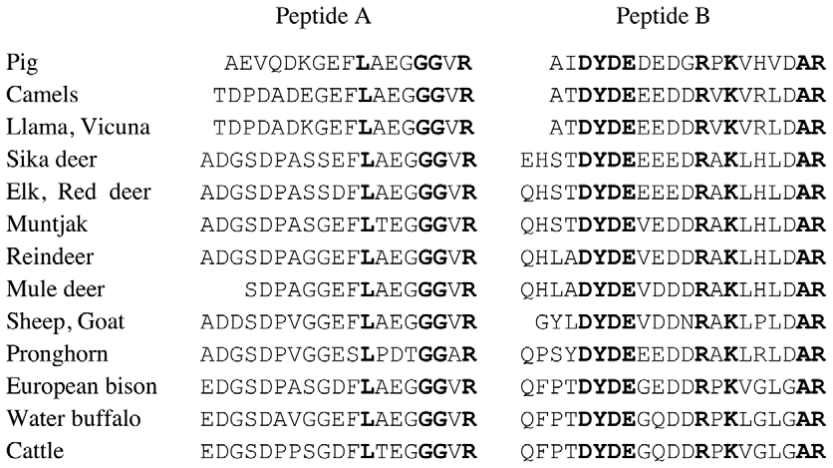
Fibrinoeptides A and B from 18 artiodactyls as determined in the mid-1960s. Fully conserved residues are bolded. Terminal glutamines (Q) in fibrinopeptides B are cyclized.

**Figure 2:**
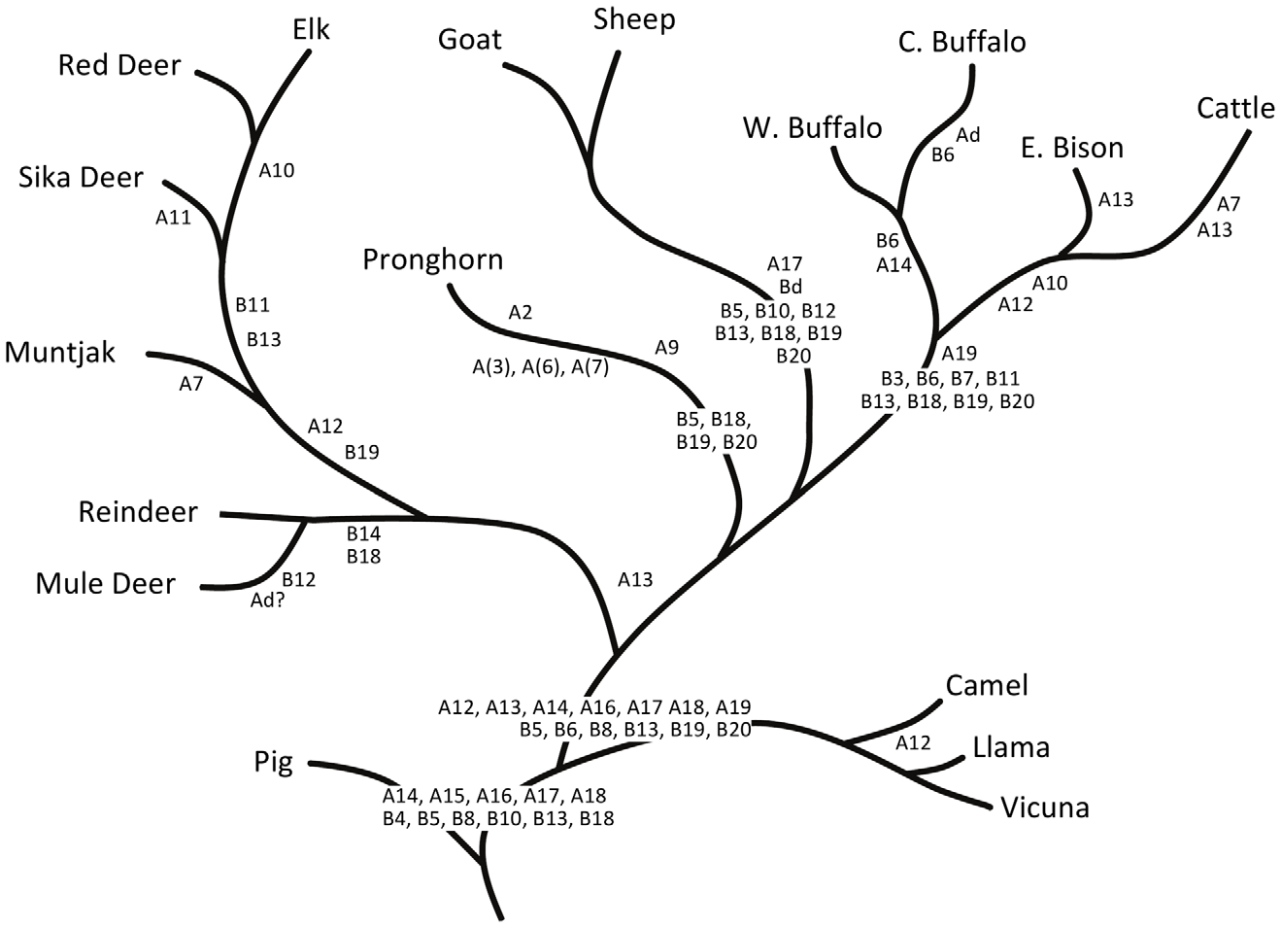
Classical relationship of artiodactyls according to G. G. Simpson (personal communication) and a mutational scheme consistent with observed fibrinopeptide A and B sequences. Residue numbering works backwards from the two carboxy-terminal arginines shown in Figure 1 (adapted from Mross and Doolittle [34]).

**Figure 3:**
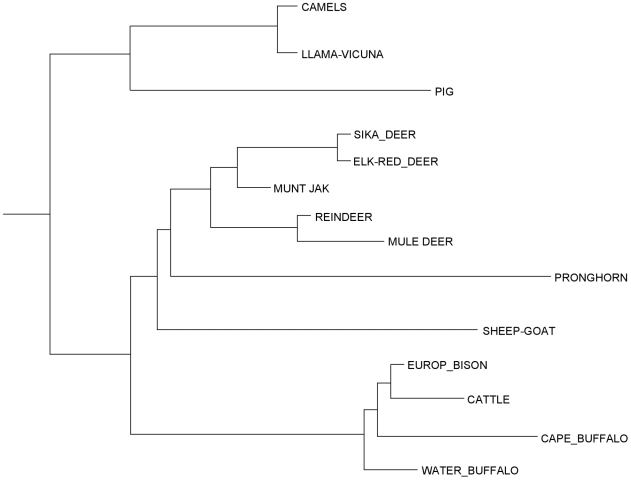
Modern computer-generated tree calculated from 1967 fibrinopeptide data shown in **Figure 1**.

Back in the United States, and, after securing a beginning faculty position, I embarked on a project to sequence, manually, as many fibrinopeptides as possible from defined groups of mammals, the goal being to reconstruct the micro-history of every amino acid substitution. By good fortune I was able to make an arrangement with the San Diego Zoo, and as a result was able to obtain the rather substantial amounts of blood from more artiodactyls, as well as other exotic creatures, that were needed to isolate fibrinopeptides [32]. Also, the problem of blocked amino-terminals in the fibrinopeptides B was overcome by isolating an enzyme that could remove cyclized glutamines [33]. The relatively small sizes of the fibrinopeptides, mostly in the range of 16–21 residues, made it possible to align the sequences by eye (Figure 1), and it was a relatively simple matter to identify the historical record of amino acid replacements and correlate them with relationships posed by classical biologists [34] (Figure 2). No computer was needed. Indeed, a belated computer analysis made recently with the same data set is quite consistent with the classical phylogeny I had obtained from Simpson (Figure 3). Nonetheless, like many others, I knew that computers were the way to go.

## Computers

In 1967, Edman made public his invention of an automatic “sequenator.” In the initial report [35], the authors reported that, beginning with only 5 mgs of myoglobin (the sequence of which was known), they had successfully identified the first 60 amino acid residues in a single run that took only four days. The machine had an ingenious design centering on a spinning cup from which various organic solvent extractions could be made. The sequenator revolutionized amino acid sequencing, and with it, protein data began to pour in.

By all accounts, the formal wedding of computers and sequence data began in the middle 1960s, not long after the first proteins longer than a hundred residues had been completed. It was then that Robert Ledley founded the National Biomedical Research Foundation (NBRF) and recruited Margaret Dayhoff and Richard Eck to edit what was intended to be an annual “atlas of sequences.” As Ledley put it in his foreword to early editions of the *Atlas of Protein Sequence and Structure*, the goal was “to collect between a single pair of covers as many as possible of the known protein and nucleotide sequences and other related data which have been educed by the scientific community” [36]. That this was thought to be possible is made clear by the fact that the first volume (1965) had only about 50 sequences, and the second volume, a year later, had only a little over a hundred, many of them only partially completed. Still, it was the beginning of an indispensable resource.

The sequences in the *Atlas* were organized functionally and according to evolutionary relationships. It also contained much more; numerous alignments were provided, as well as tables of amino acid frequencies and other interesting vitals. There were also informative chapters about the proteins and their sources. A prescient chapter in volume 3 described a mutation probability matrix for finding the evolutionary distance between two proteins [37]. It was already apparent that certain amino acid replacements were more likely than others, partly because of codon restrictions and partly attributable to the chemical nature of the amino acids involved, but the *Atlas* substitution tables went well beyond those simple notions, utilizing general frequency data and other considerations. Their model was the forerunner of numerous other substitution matrices, including still current BLOSUM tables [38].

### More Computers

Other pioneering efforts that used computers were making their mark and further setting the stage for later day bioinformatics. The classic 1967 phylogenetic tree reconstruction of cytochrome c sequences from 25 animals and fungi by Fitch and Margoliash [39] was a first order triumph. The algorithm used a simple but elegant iterative strategy.

Automatic sequence alignment methods were also being developed. Alignments of homologous proteins in the early *Atlas* volumes were made manually (the “eyeball method”), gaps being inserted in sequences whenever it seemed reasonable for maintaining the alignment. In 1970, the elegant Needleman–Wunsch algorithm for weighting gaps and gap penalties appeared [40]. From then on, computers were solidly in harness in the area of sequence comparison. The days of hand-written alignments were history.

### Ever More Sequences

The last full volume of the *Atlas* appeared in 1972 [41], after which a series of supplements appeared periodically in an effort to keep up (1973, 1976, 1978). By 1978, a magnetic tape could be purchased from the NBRF (for US$50) that contained the entire NBRF sequence collection. The total number of protein sequences from all species amounted to 1,069, representing 310 proteins and peptides, including 106 cytochrome c entries, 124 immunoglobulins, 71 hemoglobins, and 78 fibrinopeptides. Judged by modern standards, the data were biased: by kinds of protein, by organism, and by size, all a reflection of what kinds of sequence could be determined in those early days.

The introduction of DNA sequencing in the late 1970s meant that protein sequences were no longer restricted to availability, abundance, and size, and a more biologically representative set began to accumulate. Every issue of *Nature*, *Science*, or *Cell* had articles containing new cDNA sequences and their translated gene products. The question arose, could the staff at the NBRF continue to provide their careful editing, write their helpful chapters, and still keep up with the data in a timely fashion?

In fact, the final hard copy of the *Atlas* appeared in 1978 (volume 5, supplement III) [42]. The idea of two hard covers with all known sequences between them was history. The field of protein evolution was now thoroughly electronic.

Science as an endeavor thrives on obsolescence. The biochemical techniques of the 1950s and 1960s are now mostly forgotten. Similarly, many of the early computer efforts now seem merely quaint if not antiquated. Like their methodology, most of the pioneers have been doomed to anonymity. In this narrow remembrance I have mentioned only a few of the most remembered. If I had taken a different slant, I could have included many others.
